# Socioeconomic inequalities in adult oral health across different ethnic groups in England

**DOI:** 10.1186/s12955-019-1156-3

**Published:** 2019-05-17

**Authors:** Elsa K. Delgado-Angulo, Munisha Mangal, Eduardo Bernabé

**Affiliations:** 1 Faculty of Dentistry, Oral & Craniofacial Sciences, King’s College London, Denmark Hill Campus, Bessemer Road, London, SE5 9RS UK; 2 Facultad de Estomatología, Universidad Peruana Cayetano Heredia, Lima, Peru

**Keywords:** Ethnic groups, Socioeconomic factors, Oral health, Tooth loss, Toothache, Adult

## Abstract

**Background:**

Ethnic inequalities in oral health among British adults remain largely unexplored. This study explored the role of socioeconomic position (SEP) in explaining ethnic inequalities in oral health; and the consistency of socioeconomic inequalities in oral health across ethnic groups.

**Methods:**

Data from 45,599 adults, aged 16 years and over, who participated in the Health Survey for England were pooled across 5 years. The seven ethnic groups included were White British, Irish, Black Caribbean, Indian, Pakistani, Bangladeshi and Chinese. Edentulousness and toothache were the outcome measures. A composite measure of SEP was developed based on education, social class, income and economic activity using confirmatory factor analysis. Ethnic inequalities in oral health were assessed in logistic regression adjusting for sex, age, survey year and SEP.

**Results:**

Indian (OR: 0.55, 95%CI: 0.40–0.76), Pakistani (0.56, 0.38–0.83), Bangladeshi (0.35, 0.23–0.52) and Chinese (0.41, 0.25–0.66) were less likely to be edentulous than White British after controlling for SEP. Irish (1.22, 1.06–1.39) and Caribbean (1.37, 1.19–1.58) were more likely and Bangladeshi (0.83, 0.69–0.99) were less likely to have toothache than White British after controlling for SEP. Socioeconomic inequalities in edentulousness were consistently found across almost all ethnic groups while socioeconomic inequalities in toothache were found among White British and Irish only.

**Conclusion:**

This study shows that the role of SEP in explaining ethnic inequalities in oral health depended on the outcome being investigated. Socioeconomic inequalities in oral health among minority ethnic groups did not consistently reflect the patterns found in White British.

**Electronic supplementary material:**

The online version of this article (10.1186/s12955-019-1156-3) contains supplementary material, which is available to authorized users.

## Background

Health inequalities by race and ethnicity are found within and between countries, and have also been identified across different health outcomes [1, 2]. Despite differences in ethnic composition between developed countries [3], adults from ethnic minority groups often exhibit worse clinical and perceived oral health than the White population [4–6]. Until recently, data on ethnic inequalities in oral health in the United Kingdom (UK) have been very limited. Recent UK studies have shown that differences between minority ethnic groups and the White population depended on the oral health outcome being examined. White non-British (Eastern European and Other) had more caries experience whereas every Asian (Pakistani, Indian, Bangladeshi and Other) and Black (Caribbean, African and Other) group had less caries experience than White British [7]. In addition, all Asian groups had more teeth with periodontal pocketing whereas White East European, Black African and Bangladeshi had more teeth with loss of periodontal attachment than White British [8]. Finally, Asians reported better oral health-related quality of life than White and Black adults [9]. What these findings show is that there is no one-size-fits-all pattern to describe oral health inequalities by ethnicity.

A central finding from the above studies is that ethnic inequalities in oral health persisted after accounting for participants’ socioeconomic position (SEP), usually measured by education and/or occupational social class, in multivariable regression models [7–9]. However, SEP measures do not always have an equivalent meaning in different ethnic groups [10, 11] and they are not consistently associated with health across ethnic groups [12, 13]. Hence, some have recommended assessing multiple dimensions of SEP to fully characterise its contribution to ethnic inequalities in health, and potentially avoid any residual confounding [12–14].

Using national data from several years in England, we add to the existing literature on ethnic inequalities in oral health by providing a more detailed assessment of the interrelationship between ethnicity, SEP and oral health among adults. The aims of this study were to explore (i) the role of SEP in explaining ethnic inequalities in edentulousness and toothache, and (b) the consistency of socioeconomic inequalities in both oral health outcomes across ethnic groups.

## Methods

### Data source

The Health Survey for England (HSE) is a national survey that selects a nationally representative sample of private households annually, using two-stage stratified probability sampling [15]. Data were pooled together from HSE 1999, 2000, 2001, 2002 and 2005 to generate sufficiently large samples of ethnic minority populations. HSE response rate varied from 74 to 76% for those years. Most participants from ethnic minority groups were surveyed in 1999 (61.5%), when a boost sample was included of residents in England self-described as being of Bangladeshi, Black Caribbean, Chinese, Indian, Irish or Pakistani origin [16]. Participants who described themselves as White British formed the reference group. A second ethnic boost sample was selected in 2004, but it was discarded as oral health data were not collected.

There were 51,119 adults, aged 16 years and older, across the seven ethnic groups. Of them, 5520 were excluded because they did not complete the section on oral health. Therefore, the study sample included 45,599 adults with complete data on all relevant variables (weighted proportion: 92%).

### Variables selection

Information on two oral health outcomes, edentulousness and toothache, was collected using the same question formats across all survey years. Participants were first asked whether they still have some of their own teeth or have lost them all; and those with some remaining teeth were subsequently asked whether they had experienced any toothache in the last 6 months. The prevalence of edentulousness and toothache were the outcome measures used for this study.

Ethnicity was self-reported according to the participant’s family origins, with respondents being classified as White British, Irish, Black Caribbean, Indian, Pakistani, Bangladeshi and Chinese. They were the largest seven non-mixed ethnic minority groups living in England according to the 2001 UK census [17] and provide the largest sample sizes for analysis.

Four measures of SEP were chosen to address concerns about their applicability across different ethnic groups. They were education indicated by the highest formal educational qualification (none, basic or higher), social class based on the occupation of the household’s head (I, II, III-non-manual, III-manual, IV or V), annual equivalised household income, and current economic activity (unemployed or employed). A composite SEP measure was derived from fitting a one-factor model in confirmatory factor analysis. Education, social class, economic activity and income were assigned to a single latent construct representing SEP. Full-information maximum likelihood estimation was used to handle non-response in SEP measures. As some SEP measures were collected using categorical scales, the weighted least square method was used to estimate model parameters. Factor loadings were all significant and ranged from 0.60 to 0.76. The Comparative Fit Index (CFI) was 0.99 and the Root Mean Square Error of Approximation (RMSEA) was 0.039, suggesting the model was a good fit to the data. The SEP latent factor score was categorised into quintiles. Other variables included in the analysis were demographic characteristics (sex and age) and survey year.

### Data analysis

All analyses were conducted in Stata 15 (StataCorp LP, College Station, Texas, United States). Weights were used to account for unequal probability of selection and non-response rates. All ethnic groups were compared in terms of demographic factors and SEP using the Chi-squared test.

The role of SEP in ethnic inequalities in edentulousness was assessed in logistic regression models using the full study sample (*n* = 45,599). The modelling strategy was first to estimate the crude association of ethnicity with edentulousness (Model 1), and then gradually adjust for confounders. Model 2 was adjusted for sex, continuous age and survey year (categorical); and Model 3 was further adjusted for the composite measure of SEP. A similar set of models was fitted when exploring ethnic inequalities in toothache, although the sample was restricted to dentate adults (*n* = 40,737).

Finally, the association of the SEP with each oral health outcome was explored in stratified analysis to check the consistency of social gradients across ethnic groups. The Slope Index of Inequality (SII) and the Relative Index of Inequality (RII) were used to measure, respectively, absolute and relative socioeconomic inequality in oral health. Linear and Poisson regressions were used to estimate SII and RII, respectively, in models adjusted for sex, continuous age and survey year. In this analysis, a SII lower than zero (or a RII lower than one) indicates that the proportion of participants with edentulousness or toothache is more common among the better-off whereas a SII higher than zero (or a RII higher than one) indicates that the proportion of participants with edentulousness or toothache is more prevalent among the worse-off [18, 19].

## Results

Data from 45,599 adults (55% female) were analysed. Participants’ mean age was 48.0 years (SD: 18.3). Overall, 10.8% (95%CI: 10.5–11.2) reported being edentulous and 21.6% (95%CI: 21.1–22.2) of the 40,737 dentate adults reported experiencing toothache in the past 6 months. Participants excluded because of missing data were more likely to be male, younger and White British than those in the study sample. The sociodemographic composition of each ethnic group is presented in Table 1. All ethnic groups, but Irish, were living in lower SEP than White British adults. Bangladeshi were the most disadvantaged minority ethnic group, followed by Pakistani and Black Caribbean.

**Table 1:** Sociodemographic composition of the seven ethnic groups

	White British (*n* = 36,103)	Irish (*n* = 2400)	Caribbean (*n* = 1680)	Indian (*n* = 1834)	Pakistani (*n* = 1614)	Bangladeshi (*n* = 1251)	Chinese (*n* = 717)	*p* value^a^
Sex, %								< 0.001
Men	44.8	42.4	40.5	48.8	48.7	49.9	45.5	
Women	55.2	57.6	59.5	51.3	51.3	50.1	54.5	
Age in years								< 0.001
Mean (SD)	49.3 (17.0)	48.3 (16.9)	43.1 (17.9)	40.7 (16.5)	34.7 (14.6)	34.3 (16.4)	40.5 (16.3)	
Age groups, %								< 0.001
16–24 years	9.4	7.5	14.4	16.2	26.7	31.3	18.5	
25–34 years	15.2	16.0	20.4	24.2	29.9	28.0	15.6	
35–44 years	19.5	23.4	26.3	23.3	20.6	18.9	25.7	
45–54 years	16.7	17.6	9.7	16.0	12.2	7.6	24.0	
55–64 years	14.7	15.1	13.3	10.4	7.0	8.8	9.1	
65+ years	24.5	20.3	15.8	9.9	3.6	5.4	7.1	
SEP, %								< 0.001
Q1 (wealthiest)	21.0	23.2	9.8	21.5	7.1	2.3	17.8	
Q2	21.2	19.7	13.2	15.4	11.8	3.8	16.0	
Q3	20.4	18.2	21.9	19.1	17.7	10.6	21.6	
Q4	19.5	18.4	20.0	24.9	28.5	19.2	23.5	
Q5 (poorest)	18.0	20.6	35.1	19.0	35.0	64.1	21.1	

^a^ Chi-squared test was used to compare categorical variables and the t-test to compare continuous age

There were large ethnic and socioeconomic inequalities in adult oral health (Table 2). Indian, Pakistani, Bangladeshi and Chinese had lower odds of being edentulous than White British. These differences persisted after adjustments for sex, age, survey year and the composite measure of SEP. In the fully adjusted model, Indian, Pakistani, Bangladeshi and Chinese had, respectively, 45% (OR: 0.55, 95%CI: 0.40–0.76), 44% (0.56, 95%CI: 0.38–0.83), 65% (0.35, 95%CI: 0.23–0.52) and 59% (OR: 0.41, 95%CI: 0.25–0.66) lower odds of being edentulous than White British. A significant linear trend in prevalence of edentulousness according to SEP was also identified (*p* < 0.001). A different picture was observed for toothache, which was more common in Irish, Black Caribbean, Indian and Pakistani than White British. Differences for Indian and Pakistani became non-significant after adjustment for the composite SEP measure whereas differences for Bangladeshi became evident after that same adjustment. In the fully adjusted model, Irish and Caribbean had 1.22 (95%CI: 1.06–1.39) and 1.37 (95%CI: 1.19–1.58) times greater odds of having toothache whereas Bangladeshi had 17% (0.83, 95%CI: 0.69–0.99) lower odds of having toothache than White British. Although a significant linear trend in prevalence of toothache according to SEP quintiles was also identified (*p* < 0.001), only the odds for the poorest quintile were significantly different from those for the wealthiest quintile.

**Table 2:** The role of SEP in explaining ethnic inequalities in oral health outcomes

	Prevalence		Model 1^a^	Model 2^a^	Model 3^a^
	%	[95% CI]	OR^b^ [95% CI]	OR^b^ [95% CI]	OR^b^ [95% CI]
Regression models for edentulousness (*n* = 45,599)					
Ethnicity					
White British	11.6	[11.2–12.0]	1.00 [Reference]	1.00 [Reference]	1.00 [Reference]
Irish	12.1	[10.4–13.7]	1.05 [0.89–1.23]	1.25 [1.03–1.52]^c^	1.16 [0.94–1.41]
Black Caribbean	11.0	[9.2–12.7]	0.94 [0.78–1.12]	1.72 [1.39–2.14]^e^	1.13 [0.90–1.42]
Indian	3.9	[2.9–4.9]	0.31 [0.23–0.41]^e^	0.64 [0.47–0.87]^d^	0.55 [0.40–0.76]^e^
Pakistani	2.6	[1.7–3.5]	0.21 [0.14–0.29]^e^	0.93 [0.63–1.37]	0.56 [0.38–0.83]^d^
Bangladeshi	2.8	[1.8–3.8]	0.22 [0.15–0.32]^e^	0.80 [0.53–1.22]	0.35 [0.23–0.52]^e^
Chinese	2.9	[1.6–4.2]	0.23 [0.14–0.36]^e^	0.53 [0.34–0.85]^d^	0.41 [0.25–0.66]^e^
SEP measure					
Q1 (wealthiest)	0.9	[0.7–1.2]	1.00 [Reference]	1.00 [Reference]	1.00 [Reference]
Q2	2.5	[2.1–2.9]	2.70 [1.95–3.75]^e^	1.79 [1.28–2.50]^d^	1.80 [1.29–2.51]^d^
Q3	6.6	[5.9–7.3]	7.51 [5.54–10.16]^e^	3.72 [2.72–5.08]^e^	3.77 [2.76–5.14]^e^
Q4	15.1	[14.2–16.1]	18.91 [14.13–25.32]^e^	5.78 [4.28–7.81]^e^	5.91 [4.37–8.00]^e^
Q5 (poorest)	29.0	[27.9–30.2]	43.40 [32.55–57.88]^e^	11.81 [8.77–15.91]^e^	12.31 [9.12–16.63]^e^
Regression models for toothache (*n* = 40,737)					
Ethnicity					
White British	20.9	[20.3–21.5]	1.00 [Reference]	1.00 [Reference]	1.00 [Reference]
Irish	24.8	[22.5–27.2]	1.25 [1.10–1.42]^d^	1.21 [1.06–1.38]^d^	1.22 [1.06–1.39]^d^
Black Caribbean	29.6	[26.9–32.2]	1.59 [1.39–1.81]^e^	1.43 [1.25–1.65]^e^	1.37 [1.19–1.58]^e^
Indian	24.9	[22.5–27.4]	1.26 [1.10–1.44]^d^	1.15 [1.00–1.32]^c^	1.13 [0.98–1.30]
Pakistani	26.3	[23.7–28.9]	1.35 [1.18–1.55]^e^	1.16 [1.01–1.35]^c^	1.09 [0.93–1.26]
Bangladeshi	22.5	[19.6–25.4]	1.10 [0.93–1.30]	0.93 [0.78–1.11]	0.83 [0.69–0.99]^c^
Chinese	20.6	[17.3–23.9]	0.98 [0.80–1.20]	0.87 [0.71–1.08]	0.86 [0.69–1.06]
SEP measure					
Q1 (wealthiest)	20.8	[19.7–21.9]	1.00 [Reference]	1.00 [Reference]	1.00 [Reference]
Q2	20.5	[19.4–21.5]	0.99 [0.90–1.09]	0.99 [0.90–1.10]	0.99 [0.90–1.10]
Q3	21.9	[20.9–23.0]	1.05 [0.95–1.15]	1.06 [0.96–1.17]	1.06 [0.96–1.17]
Q4	21.6	[20.5–22.8]	1.05 [0.95–1.16]	1.10 [0.99–1.21]	1.10 [0.99–1.21]
Q5 (poorest)	24.3	[23.0–25.5]	1.21 [1.10–1.33]^e^	1.26 [1.15–1.39]^e^	1.27 [1.15–1.40]^e^

^a^ Model 1 reports the unadjusted associations of ethnicity and the composite measure of SEP with each oral health outcome. Model 2 included ethnicity (or the composite measure of SEP), sex, continuous age and dummy variables for survey years as explanatory variables. Model 3 included both ethnicity and the composite measure of SEP (mutually adjusted) as well as sex, continuous age and dummy variables for survey years as explanatory variables

^b^ Logistic regression was fitted and odds ratios (OR) reported

^c^*p* < 0.05; ^d^*p* < 0.01, ^e^*p* < 0.001

Socioeconomic inequalities in adult oral health were not consistently found across all ethnic groups (Table 3). While the SII and RII values for edentulousness and toothache were significant among White British, hey varied depending on the outcome among minority ethnic groups. For edentulousness, there were significant absolute socioeconomic inequalities across all minority ethnic groups, but relative socioeconomic inequalities were only found among Irish, Black Caribbean and Bangladeshi. Although RII values were not significant for Indian, Pakistani and Chinese, they were in the expected direction (i.e. edentulousness was more common among the worse-off). For toothache, absolute and relative socioeconomic inequalities were significant among Irish only. RII and SII values were in the opposite direction (i.e. toothache was more common among the better-off) among Indian, Pakistani and Bangladeshi, although they were not significant.

**Table 3:** Relative and absolute measures of socioeconomic inequality in oral health by ethnic groups

	RII^a^	[95% CI]	SII^a^	[95% CI]
Edentulousness				
White British	11.79	[9.76 to 14.24]^d^	24.06	[22.79 to 25.32]^d^
Irish	9.92	[5.16 to 19.07]^d^	22.03	[17.31 to 26.74]^d^
Black Caribbean	3.89	[1.32 to 11.45]^b^	17.30	[12.29 to 22.31]^d^
Indian	2.49	[0.52 to 11.93]	6.14	[2.48 to 9.81]^c^
Pakistani	2.64	[0.59 to 11.78]	3.58	[0.31 to 6.85]^b^
Bangladeshi	6.76	[1.32 to 14.74]^b^	4.64	[2.41 to 6.87]^d^
Chinese	8.52	[0.91 to 79.63]	7.83	[3.72 to 11.93]^d^
Toothache				
White British	1.29	[1.17 to 1.43]^d^	5.74	[3.64 to 7.83]^d^
Irish	1.44	[1.03 to 2.00]^b^	9.76	[1.48 to 18.05]^b^
Black Caribbean	1.13	[0.80 to 1.58]	3.63	[−6.41 to 13.68]
Indian	0.85	[0.59 to 1.22]	−3.84	[−13.00 to 5.32]
Pakistani	0.81	[0.57 to 1.16]	−5.34	[−14.92 to 4.24]
Bangladeshi	0.98	[0.54 to 1.79]	−0.04	[−12.26 to 12.18]
Chinese	1.09	[0.63 to 1.89]	2.23	[−9.51 to 13.97]

*RII* relative index of inequality, *SII* slope index of inequality

^a^ Estimates were adjusted for participants’ sex, continuous age and dummy variables for survey years

^b^*p* < 0.05; ^c^*p* < 0.01, ^d^*p* < 0.001

Analyses disaggregated by each individual SEP measure are shown in the Additional file 1. In line with the above results, clear ethnic inequalities in edentulousness and toothache remained after adjustments for either income, education, social class or economic activity. Moreover, SII values were more often found to be significant (especially for economic activity and income) than RII values.

## Discussion

This study found large ethnic inequalities in adult oral health in England. However, crude differences were not always in favour of the White population. All Asian groups (Indian, Pakistani, Bangladeshi and Chinese) were less likely to have lost all their teeth whereas Irish, Black Caribbean, Indian and Pakistani were more likely to have suffered toothache in the last 6 months than White British. The role of SEP in the association between ethnicity and oral health varied depending on the outcome being assessed; but it could be generally described as an attenuation of the magnitude of existing ethnic differences in oral health. For edentulousness, the ORs for all Asian groups were somewhat attenuated after accounting for SEP but remained significantly lower compared to White British. For toothache, the ORs for Indian and Pakistani become non-significant and those for Irish and Black Caribbean were somewhat attenuated after accounting for SEP but remained significantly higher than those for White British. Furthermore, a difference in the prevalence of toothache favouring Bangladeshi over White British was made evident only after accounting for SEP. Despite being one of the most disadvantaged minority ethnic groups, Bangladeshi adults were less likely to report both oral health outcomes than their White British counterparts. This finding suggests that better oral health outcomes in certain minority ethnic groups could be masked by relatively poorer SEP.

A second important finding of this study is that the direction and strength of the association between SEP and oral health was not similar across ethnic groups. Although it has been suggested that the ideal SEP measure should behave comparably across ethnic groups [20], absolute and relative measures of socioeconomic inequalities in oral health among minority ethnic groups did not consistently reflect the patterns observed in the White population. This finding implies that other forms of social disadvantage, not captured with the four SEP measured used here, might play some role in ethnic inequalities in oral health. Interestingly, socioeconomic inequalities were more consistently found with edentulousness than toothache. The latter outcome was measured among dentate adults only, which by default excludes from the analysis those with the worst oral health (edentate). This approach might explain the shape of the SEP-toothache association found in this study, which resembled a threshold effect (only adults in the poorest SEP quintile were significantly different from those in other quintiles) rather than the expected gradient. An alternative explanation is the different timeframe of both outcomes. While edentulousness measures lifetime prevalence (cumulative history of oral diseases), toothache measures period prevalence (current unmet oral healthcare needs).

The present findings reinforce concerns about SEP measures not being equally applicable to all ethnic groups [10, 11]. Social inequalities were more commonly found when economic activity or household income were chosen to represent participants’ SEP. A SEP measure should be chosen according to its suitability for the ethnic groups and health outcomes being investigated. For our main analysis, we used CFA to develop a composite SEP measure. It was an efficient way to summarise common variation in four conventional SEP measures while also using all the available information under the assumption that responses were missing at random. Latent SEP variables have been previously used to explore socioeconomic inequalities in health [21, 22] and oral health [23, 24]. The composite SEP measure was suitable to explore the interplay of ethnicity and SEP with edentulousness, but alternative measures might be needed to explore their interplay with toothache. Previous studies have suggested that the addition of an index of living standards [25] and asset-based measures [13, 14] might improve the ability to account for SEP differences between ethnic groups. However, whether that is also the case for oral health outcomes awaits confirmation. A common area-level SEP measure, the index of multiple deprivation, has been criticised because the meaning attached to living in a deprived area may vary across ethnic groups [12], partly due to the higher concentration of people from the same ethnic group in a given area [26].

Some limitations of this study need to be addressed. First, this study was based on cross-sectional data, and as such, only able to test for associations. Second, we used data collected in the early 2000’s. HSE 1999–2005 remains the most recent survey, including an ethnic boost sample and oral health data, available in England. Moreover, HSE recruits a nationally representative sample every year, thus giving us an opportunity to test our hypotheses with national data. Although the 2009 Adult Dental Health Survey (ADHS) also collected data on ethnicity, there were few participants from ethnic minority groups which limited comparisons by ethnicity [27]. That said, even though it is unlikely that ethnic and socioeconomic inequalities in oral health had changed in such a short time, data from emerging minority ethnic groups in England, such as Black African and Eastern European, could not be included. Third, the study sample represented 92% of all adult participants in seven ethnic groups. There were demographic differences between the study sample and those excluded due to missing data. Thus, the findings may not be fully generalisable to the study population. Fourth, information on edentulousness was collected through self-reports. However, earlier studies have shown that self-reported tooth counts are a valid measure of the number of remaining teeth [28, 29]. In addition, the prevalence of edentulousness was very similar to that reported in the 2009 Adult Dental Health Survey in England, which provided strong support for its validity in this sample. Finally, estimates of absolute and relative inequalities had broad confidence intervals. This is a known limitation of using summary measures of inequalities like the SII and RII, for which the estimation of confidence intervals depends on the number of SEP groups being analysed [30, 31].

## Conclusions

There were large ethnic inequalities in adult oral health, although they did not always favour the White British population. The role of SEP in explaining ethnic inequalities in oral health was dependent on the outcome being assessed. Socioeconomic inequalities in oral health among minority ethnic groups did not consistently reflect the patterns seen in White British, suggesting that other forms of social disadvantage might play some role in ethnic inequalities in oral health.

## Additional file

Additional file 1: **Table S1.** The role of individual SEP measures in explaining ethnic inequalities in edentulousness. **Table S2.** The role of individual SEP measures in explaining ethnic inequalities in experiencing toothache in the last 6 months. **Table S3.** Relative and absolute measures of socioeconomic inequality in edentulousness by ethnic groups. **Table S4.** Relative and absolute measures of socioeconomic inequality in toothache by ethnic groups. (DOCX 59 kb)

## Abbreviations

ADHS: Adult dental health survey
CFA: Confirmatory factor analysis
CFI: Comparative fit index
CI: Confidence interval
HSE: Health survey for England
OR: Odds ratio
RII: Relative index of inequality
RMSEA: Root mean square error of approximation
SD: Standard deviation
SEP: Socioeconomic position
SII: Slope index of inequality
UK: United Kingdom
