# Supplementary material for: Socioeconomic inequalities in adult oral health across different ethnic groups in England
Source: Health Qual Life Outcomes. 2019 May 17;17:85. doi: 10.1186/s12955-019-1156-3 (PMC6525386; doi:10.1186/s12955-019-1156-3)
Supplement: Supplementary file 1 — Table S1. The role of individual SEP measures in explaining ethnic inequalities in edentulousness. Table S2. The role of individual SEP measures in explaining ethnic inequalities in experiencing toothache in the last 6 months. Table S3. Relative and absolute measures of socioeconomic inequality in edentulousness by ethnic groups. Table S4. Relative and absolute measures of socioeconomic inequality in toothache by ethnic groups. (DOCX 59 kb) [file 12955_2019_1156_MOESM1_ESM.docx]

Additional file 1: **Table S1.** The role of individual SEP measures in explaining ethnic inequalities in edentulousness

|  |  | **Prevalence** | | **Model 1**^a^ | **Model 2**^a^ | **Model 3**^a^ |
| --- | --- | --- | --- | --- | --- | --- |
|  |  | **%** | **[95% CI]** | **OR [95% CI]** | **OR [95% CI]** | **OR [95% CI]** |
| *Ethnicity* | |  |  |  |  |  |
|  | White British | 11.2 | [10.7-11.6] | 1.00 [Reference] | 1.00 [Reference] | 1.00 [Reference] |
|  | Irish | 11.2 | [9.5-12.8] | 1.00 [0.84-1.19] | 1.17 [0.95-1.45] | 1.15 [0.92-1.43] |
|  | Black Caribbean | 10.8 | [8.9-12.6] | 0.96 [0.79-1.17] | 1.83 [1.45-2.30]*** | 1.43 [1.13-1.81]*** |
|  | Indian | 3.3 | [2.3-4.4] | 0.27 [0.20-0.38]*** | 0.52 [0.36-0.75]*** | 0.44 [0.30-0.64]*** |
|  | Pakistani | 2.5 | [1.5-3.4] | 0.20 [0.14-0.30]*** | 0.87 [0.57-1.35] | 0.59 [0.38-0.90]* |
|  | Bangladeshi | 3.1 | [1.8-4.4] | 0.26 [0.17-0.39]*** | 0.97 [0.59-1.61] | 0.55 [0.34-0.90]* |
|  | Chinese | 3.0 | [1.5-4.4] | 0.24 [0.15-0.41]*** | 0.56 [0.33-0.92]*** | 0.42 [0.25-0.71]** |
| *Household income* | |  |  |  |  |  |
|  | Q1 (poorest) | 19.3 | [18.2-20.5] | 1.00 [Reference] | 1.00 [Reference] | 1.00 [Reference] |
|  | Q2 | 18.2 | [17.1-19.3] | 0.93 [0.84-1.03] | 0.79 [0.69-0.90]*** | 0.77 [0.67-0.88]*** |
|  | Q3 | 11.0 | [10.1-11.9] | 0.52 [0.46-0.58]*** | 0.52 [0.44-0.60]*** | 0.50 [0.43-0.58]*** |
|  | Q4 | 3.5 | [3.0-4.0] | 0.15 [0.13-0.18]*** | 0.27 [0.22-0.32]*** | 0.26 [0.21-0.31]*** |
|  | Q5 (highest) | 2.3 | [1.9-2.7] | 0.10 [0.08-0.12]*** | 0.20 [0.16-0.25]*** | 0.20 [0.16-0.24]*** |
| *Ethnicity* | |  |  |  |  |  |
|  | English | 11.6 | [11.2-12.0] | 1.00 [Reference] | 1.00 [Reference] | 1.00 [Reference] |
|  | Irish | 12.1 | [10.4-13.7] | 1.05 [0.89-1.23] | 1.25 [1.03-1.52]* | 1.28 [1.05-1.56]* |
|  | Black Caribbean | 11.0 | [9.3-12.8] | 0.94 [0.79-1.13] | 1.74 [1.4-2.15]*** | 1.49 [1.19-1.86]*** |
|  | Indian | 3.9 | [2.9-4.9] | 0.31 [0.23-0.41]*** | 0.64 [0.47-0.87]** | 0.63 [0.45-0.87]** |
|  | Pakistani | 2.6 | [1.7-3.5] | 0.21 [0.15-0.29]*** | 0.93 [0.63-1.38] | 0.72 [0.48-1.07] |
|  | Bangladeshi | 2.8 | [1.8-3.8] | 0.22 [0.15-0.32]*** | 0.80 [0.53-1.22] | 0.50 [0.33-0.76]** |
|  | Chinese | 2.9 | [1.6-4.2] | 0.23 [0.14-0.36]*** | 0.53 [0.34-0.85]** | 0.45 [0.28-0.74]** |
| *Education* | |  |  |  |  |  |
|  | None | 25.8 | [24.8-26.7] | 1.00 [Reference] | 1.00 [Reference] | 1.00 [Reference] |
|  | Basic | 5.0 | [4.7-5.4] | 0.15 [0.14-0.17]*** | 0.38 [0.34-0.42]*** | 0.37 [0.33-0.41]*** |
|  | Higher | 1.7 | [1.3-2.1] | 0.05 [0.04-0.06]*** | 0.14 [0.11-0.18]*** | 0.14 [0.11-0.18]*** |
| *Ethnicity* | |  |  |  |  |  |
|  | English | 11.5 | [0.2-11.1] | 1.00 [Reference] | 1.00 [Reference] | 1.00 [Reference] |
|  | Irish | 11.8 | [0.8-10.1] | 1.03 [0.88-1.22] | 1.23 [1.01-1.50]* | 1.12 [0.92-1.37] |
|  | Black Caribbean | 11.3 | [0.9-9.5] | 0.98 [0.81-1.18] | 1.78 [1.43-2.22]*** | 1.28 [1.01-1.61]* |
|  | Indian | 3.7 | [0.5-2.7] | 0.30 [0.22-0.40]*** | 0.62 [0.45-0.86]** | 0.62 [0.44-0.86]** |
|  | Pakistani | 2.5 | [0.5-1.6] | 0.20 [0.14-0.29]*** | 0.88 [0.58-1.35] | 0.74 [0.48-1.15] |
|  | Bangladeshi | 3.0 | [0.6-1.9] | 0.24 [0.16-0.35]*** | 0.88 [0.57-1.37] | 0.55 [0.35-0.86]** |
|  | Chinese | 2.9 | [0.7-1.6] | 0.23 [0.14-0.37]*** | 0.53 [0.32-0.86]** | 0.52 [0.31-0.86]* |
| *Social class* | |  |  |  |  |  |
|  | V | 26.0 | [1.3-23.5] | 1.00 [Reference] | 1.00 [Reference] | 1.00 [Reference] |
|  | IV | 16.6 | [0.6-15.5] | 0.57 [0.49-0.66]*** | 0.81 [0.66-0.98]* | 0.82 [0.67-0.99]* |
|  | III-NM | 12.9 | [0.4-12.2] | 0.42 [0.37-0.49]*** | 0.61 [0.51-0.74]*** | 0.61 [0.51-0.74]*** |
|  | III-M | 9.5 | [0.4-8.6] | 0.30 [0.25-0.35]*** | 0.26 [0.21-0.31]*** | 0.26 [0.21-0.32]*** |
|  | II | 5.8 | [0.3-5.3] | 0.18 [0.15-0.21]*** | 0.22 [0.18-0.26]*** | 0.22 [0.18-0.26]*** |
|  | I | 3.5 | [0.5-2.6] | 0.10 [0.08-0.14]*** | 0.14 [0.10-0.19]*** | 0.14 [0.10-0.19]*** |
| *Ethnicity* | |  |  |  |  |  |
|  | English | 11.6 | [11.2-12.0] | 1.00 [Reference] | 1.00 [Reference] | 1.00 [Reference] |
|  | Irish | 12.1 | [10.5-13.8] | 1.05 [0.89-1.23] | 1.25 [1.03-1.52]* | 1.27 [1.04-1.54]* |
|  | Black Caribbean | 11.0 | [9.2-12.7] | 0.94 [0.78-1.12] | 1.72 [1.39-2.14]*** | 1.66 [1.34-2.07]*** |
|  | Indian | 3.9 | [2.9-4.9] | 0.31 [0.23-0.41]*** | 0.64 [0.47-0.87]** | 0.62 [0.45-0.85]** |
|  | Pakistani | 2.6 | [1.7-3.5] | 0.21 [0.15-0.29]*** | 0.93 [0.63-1.38] | 0.82 [0.56-1.21] |
|  | Bangladeshi | 2.8 | [1.8-3.8] | 0.22 [0.15-0.32]*** | 0.80 [0.52-1.21] | 0.67 [0.44-1.02] |
|  | Chinese | 2.9 | [1.6-4.2] | 0.23 [0.14-0.36]*** | 0.53 [0.34-0.85]** | 0.52 [0.32-0.83]** |
| *Economic activity* | |  |  |  |  |  |
|  | Unemployed | 20.4 | [19.7-21] | 1.00 [Reference] | 1.00 [Reference] | 1.00 [Reference] |
|  | Employed | 2.5 | [2.2-2.7] | 0.10 [0.09-0.11]*** | 0.56 [0.48-0.64]*** | 0.55 [0.47-0.64]*** |

^a^ Model 1 reports the unadjusted associations of ethnicity and the individual measure of SEP with edentulousness. Model 2 included ethnicity (or the individual measure of SEP), sex, continuous age and dummy variables for survey years as explanatory variables. Model 3 included both ethnicity and the individual measure of SEP (mutually adjusted) as well as sex, continuous age and dummy variables for survey years as explanatory variables.

^b^ Logistic regression was fitted and odds ratios (OR) reported.

*p<0.05; **p<0.01, ***p<0.001

Additional file 1: **Table S2.** The role of individual SEP measures in explaining ethnic inequalities in experiencing toothache in the last 6 months

|  |  | **Prevalence** | | **Model 1**^a^ | **Model 2**^a^ | **Model 3**^a^ |
| --- | --- | --- | --- | --- | --- | --- |
|  |  | **%** | **[95% CI]** | **OR [95% CI]** | **OR [95% CI]** | **OR [95% CI]** |
| *Ethnicity* | |  |  |  |  |  |
|  | White British | 21.4 | [0.3-20.7] | 1.00 [Reference] | 1.00 [Reference] | 1.00 [Reference] |
|  | Irish | 23.7 | [1.3-21.2] | 1.14 [0.99-1.32] | 1.12 [0.97-1.29] | 1.12 [0.97-1.30] |
|  | Black Caribbean | 30.1 | [1.5-27.2] | 1.59 [1.37-1.83]*** | 1.45 [1.25-1.69]*** | 1.38 [1.18-1.60]*** |
|  | Indian | 25.5 | [1.5-22.6] | 1.26 [1.08-1.48]** | 1.17 [1.00-1.38] | 1.12 [0.95-1.33] |
|  | Pakistani | 25.4 | [1.6-22.3] | 1.25 [1.06-1.48]** | 1.11 [0.93-1.32] | 0.99 [0.82-1.19] |
|  | Bangladeshi | 23.3 | [1.8-19.8] | 1.12 [0.92-1.37] | 0.97 [0.78-1.19] | 0.82 [0.66-1.01] |
|  | Chinese | 21.3 | [1.9-17.6] | 1.00 [0.80-1.25] | 0.92 [0.72-1.1ave 6] | 0.87 [0.69-1.10] |
| *Household income* | |  |  |  |  |  |
|  | Q1 (poorest) | 25.9 | [0.7-24.6] | 1.00 [Reference] | 1.00 [Reference] | 1.00 [Reference] |
|  | Q2 | 22.7 | [0.7-21.4] | 0.84 [0.76-0.93]*** | 0.88 [0.79-0.97]*** | 0.87 [0.78-0.97]* |
|  | Q3 | 21.8 | [0.6-20.5] | 0.79 [0.72-0.88]*** | 0.83 [0.75-0.92]*** | 0.82 [0.74-0.92]*** |
|  | Q4 | 20.9 | [0.6-19.8] | 0.76 [0.68-0.84]*** | 0.77 [0.69-0.85]*** | 0.76 [0.68-0.85]*** |
|  | Q5 (highest) | 19.9 | [0.6-18.8] | 0.71 [0.64-0.79]*** | 0.72 [0.65-0.80]*** | 0.72 [0.64-0.80]*** |
| *Ethnicity* | |  |  |  |  |  |
|  | English | 20.9 | [0.3-20.4] | 1.00 [Reference] | 1.00 [Reference] | 1.00 [Reference] |
|  | Irish | 24.9 | [1.2-22.5] | 1.25 [1.1-1.43]** | 1.21 [1.06-1.38]** | 1.22 [1.07-1.39]** |
|  | Black Caribbean | 29.4 | [1.4-26.7] | 1.57 [1.38-1.8]*** | 1.42 [1.23-1.63]*** | 1.42 [1.23-1.63]*** |
|  | Indian | 25.0 | [1.3-22.6] | 1.26 [1.1-1.45]** | 1.15 [1.00-1.33]* | 1.17 [1.01-1.35]* |
|  | Pakistani | 26.3 | [1.3-23.7] | 1.35 [1.17-1.55]*** | 1.16 [1.00-1.35]* | 1.18 [1.02-1.37]* |
|  | Bangladeshi | 22.5 | [1.5-19.6] | 1.1 [0.93-1.30] | 0.93 [0.78-1.11] | 0.95 [0.80-1.14] |
|  | Chinese | 20.6 | [1.7-17.3] | 0.98 [0.8-1.21] | 0.88 [0.71-1.09] | 0.88 [0.71-1.10] |
| *Education* | |  |  |  |  |  |
|  | None | 19.8 | [0.5-18.9] | 1.00 [Reference] | 1.00 [Reference] | 1.00 [Reference] |
|  | Basic | 22.6 | [0.3-21.9] | 1.18 [1.1-1.27]*** | 1.08 [1.00-1.16] | 1.08 [1.00-1.16] |
|  | Higher | 21.2 | [0.7-19.9] | 1.09 [0.98-1.2] | 1.01 [0.91-1.11] | 1.00 [0.90-1.11] |
| *Ethnicity* | |  |  |  |  |  |
|  | English | 20.9 | [0.3-20.3] | 1.00 [Reference] | 1.00 [Reference] | 1.00 [Reference] |
|  | Irish | 24.6 | [1.2-22.2] | 1.24 [1.08-1.41]** | 1.20 [1.05-1.37]** | 1.20 [1.05-1.38]** |
|  | Black Caribbean | 29.0 | [1.4-26.3] | 1.54 [1.35-1.77]*** | 1.40 [1.21-1.61]*** | 1.38 [1.19-1.59]*** |
|  | Indian | 24.6 | [1.3-22.1] | 1.23 [1.07-1.42]** | 1.13 [0.97-1.30] | 1.12 [0.97-1.30] |
|  | Pakistani | 25.8 | [1.4-23.0] | 1.31 [1.13-1.52]*** | 1.14 [0.97-1.34] | 1.13 [0.96-1.32] |
|  | Bangladeshi | 22.9 | [1.6-19.7] | 1.12 [0.93-1.35] | 0.95 [0.79-1.16] | 0.93 [0.77-1.13] |
|  | Chinese | 20.0 | [1.7-16.7] | 0.95 [0.77-1.17] | 0.84 [0.68-1.05] | 0.84 [0.68-1.05] |
| *Social class* | |  |  |  |  |  |
|  | V | 22.9 | [1.3-20.3] | 1.00 [Reference] | 1.00 [Reference] | 1.00 [Reference] |
|  | IV | 22.4 | [0.7-21.1] | 0.97 [0.82-1.14] | 0.93 [0.79-1.10] | 0.93 [0.79-1.10] |
|  | III-NM | 22.4 | [0.5-21.4] | 0.97 [0.83-1.13] | 0.95 [0.81-1.11] | 0.96 [0.82-1.12] |
|  | III-M | 22.4 | [0.7-21.1] | 0.97 [0.82-1.15] | 0.96 [0.81-1.13] | 0.96 [0.82-1.14] |
|  | II | 20.3 | [0.5-19.4] | 0.86 [0.73-1.00] | 0.85 [0.73-0.99]* | 0.86 [0.73-1.00] |
|  | I | 20.5 | [1.0-18.6] | 0.87 [0.72-1.05] | 0.86 [0.71-1.04] | 0.87 [0.72-1.05] |
| *Ethnicity* | |  |  |  |  |  |
|  | English | 20.9 | [0.3-20.3] | 1.00 [Reference] | 1.00 [Reference] | 1.00 [Reference] |
|  | Irish | 24.8 | [1.2-22.5] | 1.25 [1.10-1.42]** | 1.21 [1.06-1.38]** | 1.22 [1.06-1.39]** |
|  | Black Caribbean | 29.5 | [1.4-26.9] | 1.59 [1.39-1.81]*** | 1.43 [1.25-1.65]*** | 1.42 [1.23-1.63]*** |
|  | Indian | 25.0 | [1.3-22.5] | 1.26 [1.10-1.45]** | 1.15 [1.00-1.33] | 1.14 [0.99-1.32] |
|  | Pakistani | 26.3 | [1.3-23.7] | 1.35 [1.17-1.55]*** | 1.16 [1.00-1.35]* | 1.13 [0.97-1.31] |
|  | Bangladeshi | 22.4 | [1.5-19.5] | 1.09 [0.92-1.30] | 0.93 [0.78-1.11] | 0.89 [0.74-1.07] |
|  | Chinese | 20.6 | [1.7-17.3] | 0.98 [0.80-1.21] | 0.88 [0.71-1.08] | 0.87 [0.70-1.07] |
| *Economic activity* | |  |  |  |  |  |
|  | Unemployed | 21.9 | [0.4-21.1] | 1.00 [Reference] | 1.00 [Reference] | 1.00 [Reference] |
|  | Employed | 21.6 | [0.3-20.9] | 0.98 [0.93-1.04] | 0.90 [0.85-0.96]** | 0.90 [0.85-0.96]** |

^a^ Model 1 reports the unadjusted associations of ethnicity and the individual measure of SEP with toothache. Model 2 included ethnicity (or the individual measure of SEP), sex, continuous age and dummy variables for survey years as explanatory variables. Model 3 included both ethnicity and the individual measure of SEP (mutually adjusted) as well as sex, continuous age and dummy variables for survey years as explanatory variables.

^b^ Logistic regression was fitted and odds ratios (OR) reported.

*p<0.05; **p<0.01, ***p<0.001

Additional file 1: **Table S3.** Relative and absolute measures of socioeconomic inequality in edentulousness by ethnic groups

|  |  | **RII** | **[95% CI]** | **SII** | **[95% CI]** |
| --- | --- | --- | --- | --- | --- |
| Income | White British | 4.81 | [4.07 to 5.69]*** | 18.11 | [16.78 to 19.45]*** |
|  | Irish | 3.93 | [2.29 to 6.74]*** | 14.79 | [10.14 to 19.44]*** |
|  | Black Caribbean | 1.73 | [0.87 to 3.44] | 11.25 | [6.60 to 15.91]*** |
|  | Indian | 3.30 | [0.56 to 19.45] | 4.05 | [0.41 to 7.69]* |
|  | Pakistani | 0.94 | [0.25 to 3.54] | 0.73 | [-2.44 to 3.89] |
|  | Bangladeshi | 1.11 | [0.16 to 7.81] | 1.78 | [-2.42 to 5.99] |
|  | Chinese | 15.18 | [1.33 to 172.9]* | 7.84 | [3.41 to 12.26]** |
| Education | White British | 6.05 | [5.09 to 7.18]*** | 21.67 | [20.05 to 23.30]*** |
|  | Irish | 5.39 | [2.89 to 10.08]*** | 18.30 | [12.34 to 24.26]*** |
|  | Black Caribbean | 4.95 | [1.59 to 15.44]** | 20.08 | [12.37 to 27.79]*** |
|  | Indian | 1.94 | [0.62 to 6.07] | 4.59 | [0.65 to 8.52]* |
|  | Pakistani | 3.89 | [0.7 to 21.76] | 3.55 | [-0.07 to 7.17] |
|  | Bangladeshi | 2.86 | [0.37 to 21.91] | -0.21 | [-2.37 to 1.96] |
|  | Chinese | 1.77 | [0.07 to 43.03] | 3.97 | [-1.47 to 9.41] |
| Social class | White British | 4.11 | [3.62 to 4.66]*** | 17.22 | [15.84 to 18.60]*** |
|  | Irish | 4.68 | [3.05 to 7.18]*** | 17.81 | [12.55 to 23.07]*** |
|  | Black Caribbean | 2.26 | [1.19 to 4.28]* | 12.82 | [7.11 to 18.52]*** |
|  | Indian | 1.24 | [0.50 to 3.05] | 0.71 | [-2.39 to 3.81] |
|  | Pakistani | 2.19 | [0.55 to 8.67] | 2.96 | [-0.81 to 6.73] |
|  | Bangladeshi | 3.02 | [0.95 to 9.57] | 3.96 | [0.62 to 7.30]* |
|  | Chinese | 1.89 | [0.22 to 16.33] | 4.36 | [-0.57 to 9.28] |
| Economic activity | White British | 3.90 | [2.92 to 5.20]*** | 15.93 | [14.52 to 17.33]*** |
|  | Irish | 3.55 | [1.17 to 10.78]* | 17.69 | [11.55 to 23.82]*** |
|  | Black Caribbean | 2.64 | [0.82 to 8.54] | 18.62 | [13.48 to 23.75]*** |
|  | Indian | 5.44 | [0.44 to 67.74] | 10.26 | [6.23 to 14.29]*** |
|  | Pakistani | 1.51 | [0.26 to 8.85] | 3.46 | [-0.32 to 7.25] |
|  | Bangladeshi | 5.86 | [0.73 to 47.07] | 6.22 | [3.05 to 9.40]*** |
|  | Chinese | 6.60 | [0.12 to 36.15] | 10.50 | [5.34 to 15.65]*** |

RII: relative index of inequality; SII: slope index of inequality

^a^ Estimates were adjusted for participants’ sex, continuous age and dummy variables for survey years.

*p<0.05; **p<0.01, ***p<0.001

Additional file 1: **Table S4.** Relative and absolute measures of socioeconomic inequality in toothache by ethnic groups

|  |  | **RII** | **[95% CI]** | **SII** | **[95% CI]** |
| --- | --- | --- | --- | --- | --- |
| Income | White British | 1.39 | [1.25 to 1.54]*** | 7.32 | [5.07 to 9.57]*** |
|  | Irish | 1.44 | [1.00 to 2.09] | 9.21 | [0.43 to 18.00]* |
|  | Black Caribbean | 1.15 | [0.81 to 1.62] | 4.23 | [-6.15 to 14.60] |
|  | Indian | 0.89 | [0.57 to 1.38] | -2.80 | [-13.82 to 8.22] |
|  | Pakistani | 0.95 | [0.62 to 1.47] | -1.23 | [-12.30 to 9.85] |
|  | Bangladeshi | 0.69 | [0.33 to 1.45] | -7.61 | [-25.62 to 10.39] |
|  | Chinese | 0.84 | [0.44 to 1.59] | -3.68 | [-17.56 to 10.20] |
| Education | White British | 1.00 | [0.89 to 1.12] | 0.02 | [-2.28 to 2.32] |
|  | Irish | 0.88 | [0.61 to 1.26] | -2.94 | [-11.85 to 5.97] |
|  | Black Caribbean | 0.96 | [0.63 to 1.46] | -1.10 | [-13.31 to 11.11] |
|  | Indian | 0.94 | [0.66 to 1.36] | -1.17 | [-10.43 to 8.09] |
|  | Pakistani | 0.70 | [0.48 to 1.03] | -9.18 | [-19.63 to 1.27] |
|  | Bangladeshi | 0.72 | [0.41 to 1.29] | -6.84 | [-18.84 to 5.15] |
|  | Chinese | 1.08 | [0.55 to 2.14] | 1.95 | [-12.31 to 16.22] |
| Social class | White British | 1.14 | [1.03 to 1.26]** | 2.76 | [0.74 to 4.79]** |
|  | Irish | 1.60 | [1.13 to 2.26]** | 11.55 | [3.03 to 20.07]** |
|  | Black Caribbean | 0.86 | [0.61 to 1.23] | -4.19 | [-14.42 to 6.04] |
|  | Indian | 0.96 | [0.66 to 1.42] | -0.85 | [-10.25 to 8.55] |
|  | Pakistani | 0.96 | [0.66 to 1.40] | -0.93 | [-10.67 to 8.81] |
|  | Bangladeshi | 0.74 | [0.45 to 1.21] | -6.21 | [-17.77 to 5.35] |
|  | Chinese | 0.69 | [0.39 to 1.22] | -7.30 | [-18.89 to 4.28] |
| Economic activity | White British | 1.21 | [1.08 to 1.36]** | 4.75 | [2.27 to 7.24]*** |
|  | Irish | 1.20 | [0.80 to 1.80] | 5.66 | [-4.79 to 16.10] |
|  | Black Caribbean | 1.32 | [0.92 to 1.90] | 8.40 | [-2.35 to 19.15] |
|  | Indian | 0.89 | [0.60 to 1.33] | -2.38 | [-12.48 to 7.72] |
|  | Pakistani | 0.88 | [0.56 to 1.37] | -2.75 | [-14.13 to 8.63] |
|  | Bangladeshi | 1.02 | [0.49 to 2.09] | 1.42 | [-12.58 to 15.43] |
|  | Chinese | 1.23 | [0.65 to 2.32] | 4.91 | [-8.48 to 18.30] |

RII: relative index of inequality; SII: slope index of inequality

^a^ Estimates were adjusted for participants’ sex, continuous age and dummy variables for survey years.

*p<0.05; **p<0.01, ***p<0.001
